# Antimicrobial Susceptibility Testing Performed in RPMI 1640 Reveals Azithromycin Efficacy against Carbapenem-Resistant Acinetobacter baumannii and Predicts *In Vivo* Outcomes in Galleria mellonella

**DOI:** 10.1128/aac.01320-22

**Published:** 2022-12-05

**Authors:** Sarah Miller, Kristine Goy, Rosemary She, Brad Spellberg, Brian Luna

**Affiliations:** a Department of Molecular Microbiology and Immunology, Keck School of Medicine at USC, Los Angeles, California, USA; b Department of Pathology, Keck School of Medicine at USC, Los Angeles, California, USA; c Los Angeles County-USC Medical Center, Los Angeles, California, USA

**Keywords:** antibiotic resistance, *Acinetobacter baumannii*, azithromycin, colistin

## Abstract

Antimicrobial susceptibility testing (AST) in RPMI 1640, a more physiologically relevant culture medium, revealed that a substantial proportion of carbapenem-resistant Acinetobacter baumannii isolates were susceptible to azithromycin, a macrolide antibiotic not currently considered effective against A. baumannii. Experiments using Galleria mellonella validated these *in vitro* data. Our finding that RPMI 1640’s predictive accuracy for *in vivo* outcomes is superior to that of Mueller-Hinton II broth also supports the use of more physiologically relevant AST culturing conditions.

## INTRODUCTION

Increasing resistance to important antibiotics like carbapenems and last-resort drugs such as colistin is especially of concern and has led the U.S. Centers for Disease Control to list carbapenem-resistant Acinetobacter baumannii as an urgent threat in its 2019 *Antibiotic Resistance Threats Report* (1).

Recent *in vitro* studies using traditional culturing methods have suggested that colistin (COL) resistance imparts A. baumannii with azithromycin (AZM) susceptibility, despite macrolides being considered largely ineffective against the species (2, 3). AZM is the second most frequently prescribed antibiotic in the United States, has a broad spectrum of activity, and has favorable safety characteristics (4, 5). Macrolides are traditionally thought active against atypical pneumonia agents (6) but not against nonfermenting Gram-negative pathogens such as A. baumannii. Therefore, no clinical laboratory standards exist for evaluating AZM activity against A. baumannii (7).

Our group previously characterized rifabutin’s iron-dependent efficacy against A. baumannii using more physiologically relevant RPMI 1640 mammalian culture medium, which does contain a physiologically normal concentration of bicarbonate, to perform antimicrobial susceptibility testing (AST) (8, 9). AZM’s mechanism of entry into bacterial cells depends on transmembrane proton motive force, powered by bicarbonate, which is present in the host environment and RPMI 1640 but absent from Mueller-Hinton II (MHII) medium (10, 11). We therefore evaluated AZM activity against carbapenem-resistant A. baumannii clinical isolates in RPMI 1640 versus MHII media.

### MIC distributions.

It was not previously known how broadly active AZM was against carbapenem-resistant A. baumannii isolates, as no single study has tested more than 6 unique clinical isolates (2, 12, 13). Therefore, we determined the AZM MICs against a larger panel of 77 carbapenem-resistant A. baumannii clinical isolates, using the broth microdilution method per the Clinical and Laboratory Standards Institute (CLSI) in either MHII or RPMI 1640 as the culture medium (7–9, 14).

**FIG 1:**
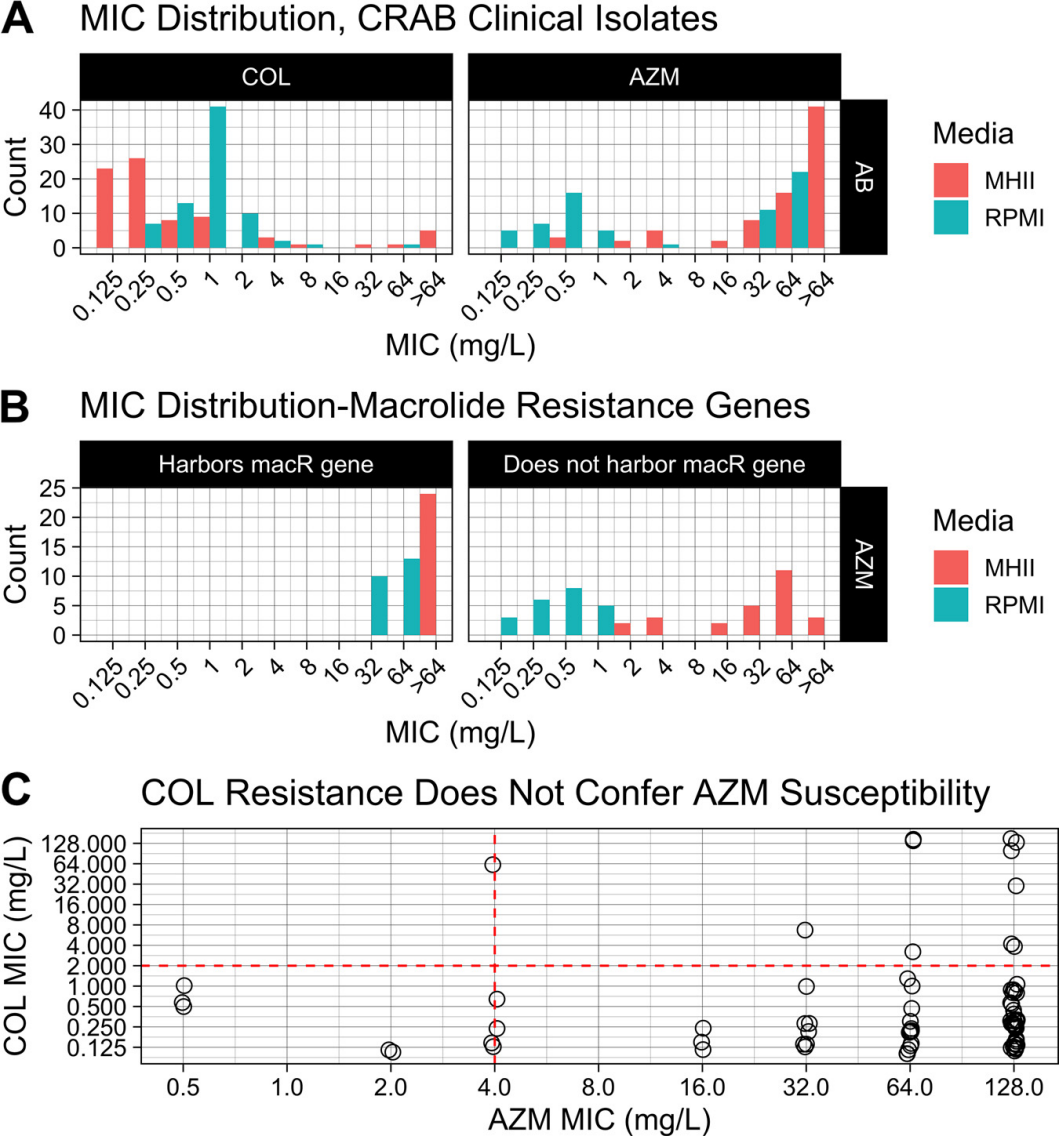
MIC distributions and relationship between colistin (COL) resistance and azithromycin (AZM) susceptibility. Dashed lines delineate the CLSI susceptible breakpoints for each respective drug. (A) MICs for both COL (left) and AZM (right) were determined against 77 carbapenem-resistant A. baumannii (CRAB) clinical isolates using either MHII or RPMI 1640 media. The MHII and RPMI data points lie to the left or right, respectively, of their assigned *x* axis tick mark. (B) Per the CDC-FDA AR Isolate Bank, the sequenced strains tested here harbored either both the macrolide resistance genes *mphE* and *msrE* or neither. The panel labeled “Harbors macR gene” includes only strains that harbor both *mphE* and *msrE* (*n* = 50 carbapenem-resistant clinical isolates for MHII; *n* = 45 carbapenem-resistant clinical isolates for RPMI). (C) MICs performed in traditional MHII medium (*n* = 81 clinical isolates; 77 carbapenem-resistant and 4 carbapenem-susceptible isolates).

Because the AZM susceptibility breakpoints have not been determined by CLSI for A. baumannii, we used the AZM breakpoints established for H. influenzae, a Gram-negative coccobacillus (7). There was a significant difference in the distribution of the AZM MICs in MHII versus RPMI media (*P* < 1.24E–10; Mann-Whitney) (Fig. 1A). Of the 77 total isolates, 34 (47% of the 72 capable of growing in RPMI) were considered AZM susceptible (MIC, ≤4 mg/L) in RPMI, whereas only 10 (13% of 77) were susceptible in MHII (Fig. 1A). Select isolates’ inability to grow in RPMI is unsurprising, given that previous reports have found that iron-limited environments, such as that in RPMI, can interfere with A. baumannii’s ability to grow (15).

Of these 77 isolates, 50 were already sequenced by the CDC-FDA AR Isolate Bank for known resistance genes. Within this 50-isolate subpopulation, 24 (48%) harbored the known macrolide resistance genes *mphE* and *msrE*, and all of these isolates contained both *mphE* and *msrE*. Per the Comprehensive Antibiotic Resistance Database, *mphE* and *msrE* are prevalent in 38.12% and 38.44%, respectively, of sequenced A. baumannii whole genomes (*n* = 5,422) submitted to NCBI Genomes and IslandViewer (16). Thus, our panel is a fair representation of the greater population of A. baumannii clinical isolates and was not biased. Of these 24 isolates harboring a macrolide resistance gene, none (0% of the 23 capable of growing in RPMI) were susceptible to AZM in either MHII or RPMI media (Fig. 1B). Of the remaining 26 isolates that did not harbor known macrolide resistance genes, 5 and 22 (19% of 26 and 100% of the 22 isolates capable of growing in RPMI) were susceptible to AZM in MHII and RPMI media, respectively (Fig. 1B). While previous reports suggested that COL resistance in MHII medium might correlate with A. baumannii susceptibility to AZM in MHII, this was not true among the strains we tested (Fig. 1C) (2, 3). We instead found that COL resistance was more associated with AZM resistance in the limited number of COL-resistant isolates included here (Fig. 1C).

### *In vivo* AZM efficacy.

To determine whether MIC testing in MHII or RPMI media was more predictive of *in vivo* drug efficacy, we selected 6 clinical isolates at random to be used in a Galleria mellonella infection model: 3 isolates resistant to AZM in both MHII and RPMI media and 3 isolates resistant to AZM in MHII medium but susceptible to AZM in RPMI medium (17–21). Larvae weighing 210 to 270 mg were disinfected with 70% ethanol and infected subcutaneously using a syringe pump with phosphate-buffered saline (PBS) as a control or 10 μL A. baumannii suspension to determine the respective 100% lethal dose (LD_100_) values. In subsequent experiments, larvae were infected with the previously determined LD_100_ CFU of each strain and treated with 10 μL PBS or 5, 15, 50, 150, or 450 mg/kg AZM 1 h postinfection. No infection control larvae received 2 doses of 10 μL PBS. For all experiments, larvae were incubated in 100-mm petri dishes at 37°C, and survival was monitored up to day 4 postinfection.

**FIG 2:**
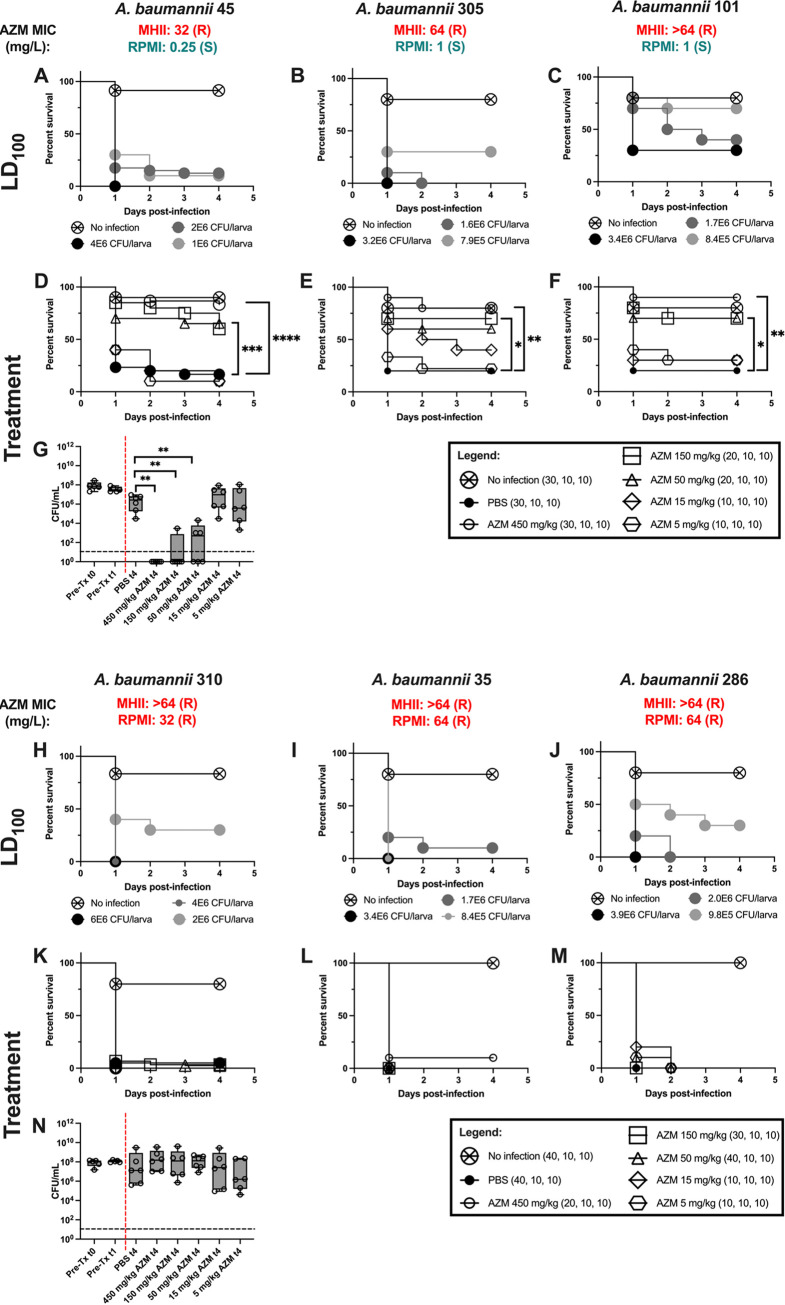
*In vivo* AZM efficacy. (A to C, H to J) Galleria mellonella larvae were challenged with various bacterial inocula to determine strain-specific LD_100_ values. (D to F, K to M) To determine AZM efficacy, Galleria mellonella larvae were infected with lethal inoculum using A. baumannii strains 45 (1.90E6 to 2.08E6 CFU/larva), 305 (3.33E6 CFU/larva), 101 (3.90E6 CFU/larva), 310 (4.05E6 to 6.15E6 CFU/larva), 35 (2.95E6 CFU/larva), or 286 (2.20E6 CFU/larva) before being treated with PBS or AZM. No infection control received 2 doses of PBS. Statistical comparisons were made using the log-rank (Mantel-Cox) test to compare survival between PBS-treated and each AZM-treated group; *, *P* ≤ 0.05; **, *P* ≤ 0.01; ***, *P* ≤ 0.001; ****, *P* ≤ 0.0001. N values in parentheses are in order of A. baumannii strains 45, 305, and 101 (top) or A. baumannii strains 310, 35, and 286 (bottom) and are nonuniform across groups because multiple experiments testing different dosages are represented in the figure. (G, N) Survival experiments were performed as described, and hemolymph was collected and plated at 0 h postinfection (PI) before treatment (t0), 1 h PI before treatment (t1), and 4 h PI after treatment (t4). Tx, treatment. Statistical comparisons were made using the Mann-Whitney test to compare the CFU burden between the PBS-treated group and each AZM-treated group at 4 h PI. The CFU limit of detection (1E1 CFU/mL) is delineated by a horizontal black line. Pretreatment and posttreatment groups are separated by a vertical red line (*n* = 5 for each pretreatment group; *n* = 6 for each posttreatment group).

A. baumannii 45, 305, and 101 were resistant to AZM in MHII medium (32, 64, and >64 mg/L, respectively) but appeared susceptible in RPMI medium (0.25, 1, and 1 mg/L, respectively). In contrast, A. baumannii 310, 35, and 286 were resistant in both MHII medium (>64 mg/L) and RPMI medium (32, 64, and 64 mg/L, respectively) (Fig. 2). If MHII medium were more predictive of *in vivo* outcomes, then we would expect no strain to respond to treatment with AZM; however, should RPMI be more predictive, then we would expect the RPMI-susceptible A. baumannii isolates but not the RPMI-resistant ones to respond to treatment. AZM treatment significantly improved the survival of the Galleria mellonella larvae that were infected with A. baumannii RPMI-susceptible isolates, but not the RPMI-resistant ones, compared to the PBS control group (log-rank test: A. baumannii 45, 50 mg/kg, *P* = 0.004; 150 mg/kg, *P* = 0.003; 450 mg/kg, *P* < 0.0001; A. baumannii 305, 150 mg/kg, *P* = 0.0285; 450 mg/kg, *P* = 0.0059; A. baumannii 101, 50 mg/kg, *P* = 0.0285; 150 mg/kg, *P* = 0.0285; 450 mg/kg, *P* = 0.0022) (Fig. 2D to F). Larvae challenged with A. baumannii 310, 35, or 286 were not rescued in a statistically significant manner at any AZM dose tested (Fig. 2K to M). Importantly, MIC testing performed in RPMI medium, but not MHII medium, accurately predicted these *in vivo* survival outcomes (Fig. 2).

To evaluate the bacterial clearance, hemolymph from each larva was collected at 0 h, 1 h, and 4 h postinfection into its own well of a 96-well plate. The CFU from hemolymph were determined by plating serial dilutions on CHROMagar Orientation medium and incubating them at 37°C overnight. The CFU burden in Galleria mellonella larvae challenged with A. baumannii 45 (susceptible to AZM in RPMI medium) was significantly lower in groups treated with AZM at 50 mg/kg or higher than in those treated with PBS (450, 150, and 50 mg/kg, *P* = 0.0022; Mann-Whitney) (Fig. 2G). In contrast, the CFU burden in larvae infected with A. baumannii 310 (resistant to AZM in RPMI medium) and treated with AZM at any tested dose was not significantly lower than that in the PBS-treated control group (Fig. 2N). These data are consistent with the survival results.

### Conclusion.

Given the increasing rates of antibiotic resistance, the high cost of developing new antibiotics, and the years-long development timeline, it is critical that we make use most efficiently of the antibiotics that are currently available (22–24). Here, we found that AST performed in RPMI 1640 medium, but not MHII medium, revealed that a high proportion of carbapenem-resistant A. baumannii strains were susceptible to AZM. These results suggest that, based on the limited number of strains tested, AZM warrants clinical investigation and may be a promising therapy for resistant A. baumannii infections. Furthermore, these results add to a growing body of literature demonstrating that traditional susceptibility testing methods using rich media to maximize the bacterial growth may result in poor ability to predict the *in vivo* efficacy. Additional study is needed to define the potential role of AZM in the treatment of carbapenem-resistant A. baumannii infections and to define optimal *in vitro* testing methodologies that most accurately predict the *in vivo* efficacy.
