# Supplementary material for: Antimicrobial Susceptibility Testing Performed in RPMI 1640 Reveals Azithromycin Efficacy against Carbapenem-Resistant Acinetobacter baumannii and Predicts In Vivo Outcomes in Galleria mellonella
Source: Antimicrob Agents Chemother. 2022 Dec 5;67(1):e01320-22. doi: 10.1128/aac.01320-22 (PMC9872712; doi:10.1128/aac.01320-22)
Supplement: Supplemental file 1 — Table S1. Download aac.01320-22-s0001.pdf, PDF file, 0.1 MB [file aac.01320-22-s0001.pdf]

Supplemental Table 1

Strain identification

			COL				AZM			
			MHII		RPMI		MHII		RPMI	
**Strain**	**Origin**	**Harbors known macrolide resistance gene?**	**MIC (mg/L)**	**CLSI breakpoint interpretation (*A. baumannii*)**	**MIC (mg/L)**	**CLSI breakpoint interpretation (*A. baumannii* in MHII)**	**MIC (mg/L)**	**CLSI breakpoint interpretation (*H. influenzae*)**	**MIC (mg/L)**	**CLSI breakpoint interpretation (*H. influenzae* in MHII)**
33	CDC-FDA AR Isolate Bank	None	0.13	I	0.25	I	2	S	0.25	S
36	CDC-FDA AR Isolate Bank	None	0.13	I	0.5	I	64	R	No growth	
37	CDC-FDA AR Isolate Bank	None	0.13	I	0.25	I	2	S	No growth	
45	CDC-FDA AR Isolate Bank	None	0.13	I	0.5	I	32	R	0.25	S
52	CDC-FDA AR Isolate Bank	None	0.13	I	0.25	I	4	S	0.13	S
63	CDC-FDA AR Isolate Bank	None	0.13	I	0.5	I	32	R	0.5	S
70	CDC-FDA AR Isolate Bank	None	0.13	I	0.25	I	4	S	0.13	S
88	CDC-FDA AR Isolate Bank	None	0.25	I	1	I	16	R	0.13	S
101	CDC-FDA AR Isolate Bank	None	1	I	1	I	>64	R	1	S
273	CDC-FDA AR Isolate Bank	None	0.13	I	0.25	I	64	R	0.5	S
274	CDC-FDA AR Isolate Bank	None	0.25	I	1	I	16	R	0.25	S
277	CDC-FDA AR Isolate Bank	None	0.25	I	1	I	>64	R	0.5	S
281	CDC-FDA AR Isolate Bank	None	0.25	I	2	I	64	R	0.5	S
284	CDC-FDA AR Isolate Bank	None	0.25	I	1	I	>64	R	1	S
285	CDC-FDA AR Isolate Bank	None	0.25	I	1	I	64	R	0.5	S
287	CDC-FDA AR Isolate Bank	None	0.25	I	No growth		64	R	No growth	
292	CDC-FDA AR Isolate Bank	None	0.25	I	1	I	32	R	0.5	S
294	CDC-FDA AR Isolate Bank	None	0.25	I	1	I	4	S	0.5	S
295	CDC-FDA AR Isolate Bank	None	0.25	I	No growth		64	R	No growth	
296	CDC-FDA AR Isolate Bank	None	0.13	I	0.5	I	64	R	0.25	S

Supplemental Table 1

Strain identification

			COL				AZM			
			MHII		RPMI		MHII		RPMI	
**Strain**	**Origin**	**Harbors known macrolide resistance gene?**	**MIC (mg/L)**	**CLSI breakpoint interpretation (*A. baumannii*)**	**MIC (mg/L)**	**CLSI breakpoint interpretation (*A. baumannii* in MHII)**	**MIC (mg/L)**	**CLSI breakpoint interpretation (*H. influenzae*)**	**MIC (mg/L)**	**CLSI breakpoint interpretation (*H. influenzae* in MHII)**
305	CDC-FDA AR Isolate Bank	None	0.25	I	1	I	64	R	1	S
306	CDC-FDA AR Isolate Bank	None	1	I	1	I	64	R	1	S
312	CDC-FDA AR Isolate Bank	None	0.13	I	1	I	32	R	0.25	S
313	CDC-FDA AR Isolate Bank	None	0.25	I	1	I	64	R	0.5	S
56	CDC-FDA AR Isolate Bank	*mphE, msrE*	1	I	1	I	>64	R	32	R
288	CDC-FDA AR Isolate Bank	*mphE, msrE*	>64	R	1	I	>64	R	32	R
289	CDC-FDA AR Isolate Bank	None	4	R	1	I	64	R	0.5	S
303	CDC-FDA AR Isolate Bank	*mphE, msrE*	>64	R	2	I	>64	R	32	R
304	CDC-FDA AR Isolate Bank	None	8	R	0.5	I	32	R	1	S
307	CDC-FDA AR Isolate Bank	*mphE, msrE*	>64	R	1	I	>64	R	64	R
308	CDC-FDA AR Isolate Bank	*mphE, msrE*	32	R	1	I	>64	R	32	R
309	CDC-FDA AR Isolate Bank	*mphE, msrE*	16	R	2	I	>64	R	32	R
310	CDC-FDA AR Isolate Bank	*mphE, msrE*	4	R	4	R	>64	R	32	R
35	CDC-FDA AR Isolate Bank	*mphE, msrE*	0.13	I	1	I	>64	R	64	R
78	CDC-FDA AR Isolate Bank	*mphE, msrE*	0.5	I	2	I	>64	R	64	R
83	CDC-FDA AR Isolate Bank	*mphE, msrE*	0.25	I	2	I	>64	R	64	R
275	CDC-FDA AR Isolate Bank	*mphE, msrE*	0.25	I	1	I	>64	R	64	R
278	CDC-FDA AR Isolate Bank	*mphE, msrE*	0.5	I	1	I	>64	R	64	R
279	CDC-FDA AR Isolate Bank	*mphE, msrE*	0.25	I	1	I	>64	R	64	R
282	CDC-FDA AR Isolate Bank	*mphE, msrE*	0.25	I	2	I	>64	R	64	R

Supplemental Table 1

Strain identification

			COL				AZM			
			**MHII**		**RPMI**		**MHII**		**RPMI**	
**Strain**	**Origin**	**Harbors known macrolide resistance gene?**	**MIC (mg/L)**	**CLSI breakpoint interpretation (*A. baumannii*)**	**MIC (mg/L)**	**CLSI breakpoint interpretation (*A. baumannii* in MHII)**	**MIC (mg/L)**	**CLSI breakpoint interpretation (*H. influenzae*)**	**MIC (mg/L)**	**CLSI breakpoint interpretation (*H. influenzae* in MHII)**
283	CDC-FDA AR Isolate Bank	*mphE, msrE*	0.5	I	1	I	>64	R	64	R
286	CDC-FDA AR Isolate Bank	*mphE, msrE*	0.25	I	1	I	>64	R	64	R
290	CDC-FDA AR Isolate Bank	*mphE, msrE*	0.25	I	1	I	>64	R	64	R
291	CDC-FDA AR Isolate Bank	*mphE, msrE*	0.25	I	2	I	>64	R	64	R
297	CDC-FDA AR Isolate Bank	*mphE, msrE*	0.25	I	1	I	>64	R	32	R
298	CDC-FDA AR Isolate Bank	*mphE, msrE*	0.13	I	1	I	>64	R	64	R
299	CDC-FDA AR Isolate Bank	*mphE, msrE*	1	I	4	R	>64	R	No growth	
301	CDC-FDA AR Isolate Bank	*mphE, msrE*	0.13	I	1	I	>64	R	32	R
302	CDC-FDA AR Isolate Bank	*mphE, msrE*	0.5	I	2	I	>64	R	64	R
311	CDC-FDA AR Isolate Bank	*mphE, msrE*	0.13	I	1	I	>64	R	32	R
LAC4 WT	Cal State LA (Dr. Howard Xu) [Valentine '08 J Clin Micro]	Not sequenced	0.13	I	1	I	2	S	0.25	S
LAC4 ColR	Generated in-house	Not sequenced	1	I	64	R	4	S	0.5	S
HUMC1	Harbor UCLA Medical Center [Luo '12 PLoS One]	Not sequenced	0.25	I	0.5	I	>64	R	64	R
VA-Ab07	Case Western Reserve University (Dr. Robert Bonomo)	Not sequenced	0.13	I	1	I	>64	R	>64	R
VA-Ab08	Case Western Reserve University (Dr. Robert Bonomo)	Not sequenced	0.13	I	1	I	>64	R	64	R

Supplemental Table 1

Strain identification

			COL				AZM			
			MHII		RPMI		MHII		RPMI	
**Strain**	**Origin**	**Harbors known macrolide resistance gene?**	**MIC (mg/L)**	**CLSI breakpoint interpretation (*A. baumannii*)**	**MIC (mg/L)**	**CLSI breakpoint interpretation (*A. baumannii* in MHII)**	**MIC (mg/L)**	**CLSI breakpoint interpretation (*H. influenzae*)**	**MIC (mg/L)**	**CLSI breakpoint interpretation (*H. influenzae* in MHII)**
VA-Ab11	Case Western Reserve University (Dr. Robert Bonomo)	Not sequenced	>64	R	0.5	I	64	R	0.5	S
VA-Ab15	Case Western Reserve University (Dr. Robert Bonomo)	Not sequenced	>64	R	0.5	I	64	R	0.5	S
VA-Ab17	Case Western Reserve University (Dr. Robert Bonomo)	Not sequenced	1	I	1	I	>64	R	32	R
VA-Ab23	Case Western Reserve University (Dr. Robert Bonomo)	Not sequenced	0.13	I	1	I	64	R	0.5	S
VA-Ab39	Case Western Reserve University (Dr. Robert Bonomo)	Not sequenced	1	I	1	I	32	R	0.13	S
VA-Ab55	Case Western Reserve University (Dr. Robert Bonomo)	Not sequenced	1	I	0.5	I	>64	R	64	R
VA-Ab58	Case Western Reserve University (Dr. Robert Bonomo)	Not sequenced	0.25	I	0.5	I	>64	R	>64	R
VA-Ab59	Case Western Reserve University (Dr. Robert Bonomo)	Not sequenced	0.13	I	0.5	I	>64	R	>64	R

Supplemental Table 1

Strain identification

			COL				AZM			
			MHII		RPMI		MHII		RPMI	
**Strain**	**Origin**	**Harbors known macrolide resistance gene?**	**MIC (mg/L)**	**CLSI breakpoint interpretation (*A. baumannii*)**	**MIC (mg/L)**	**CLSI breakpoint interpretation (*A. baumannii* in MHII)**	**MIC (mg/L)**	**CLSI breakpoint interpretation (*H. influenzae*)**	**MIC (mg/L)**	**CLSI breakpoint interpretation (*H. influenzae* in MHII)**
VA-Ab64	Case Western Reserve University (Dr. Robert Bonomo)	Not sequenced	0.25	I	1	I	>64	R	>64	R
VA-Ab65	Case Western Reserve University (Dr. Robert Bonomo)	Not sequenced	0.25	I	1	I	>64	R	>64	R
VA-Ab68	Case Western Reserve University (Dr. Robert Bonomo)	Not sequenced	0.5	I	1	I	64	R	0.5	S
VA-Ab71	Case Western Reserve University (Dr. Robert Bonomo)	Not sequenced	0.5	I	1	I	>64	R	64	R
VA-Ab72	Case Western Reserve University (Dr. Robert Bonomo)	Not sequenced	0.25	I	2	I	32	R	0.25	S
VA-Ab73	Case Western Reserve University (Dr. Robert Bonomo)	Not sequenced	1	I	0.25	I	0.5	S	0.13	S
VA-Ab74	Case Western Reserve University (Dr. Robert Bonomo)	Not sequenced	0.5	I	8	R	0.5	S	4	S
VA-Ab75	Case Western Reserve University (Dr. Robert Bonomo)	Not sequenced	0.5	I	64	R	0.5	S	64	R

Supplemental Table 1

Strain identification

			COL				AZM			
			MHII		RPMI		MHII		RPMI	
**Strain**	**Origin**	**Harbors known macrolide resistance gene?**	**MIC (mg/L)**	**CLSI breakpoint interpretation (*A. baumannii*)**	**MIC (mg/L)**	**CLSI breakpoint interpretation (*A. baumannii* in MHII)**	**MIC (mg/L)**	**CLSI breakpoint interpretation (*H. influenzae*)**	**MIC (mg/L)**	**CLSI breakpoint interpretation (*H. influenzae* in MHII)**
VA-Ab78	Case Western Reserve University (Dr. Robert Bonomo)	Not sequenced	0.5	I	1	I	>64	R	64	R
VA-Ab81	Case Western Reserve University (Dr. Robert Bonomo)	Not sequenced	0.25	I	0.5	I	32	R	0.5	S
VA-Ab85	Case Western Reserve University (Dr. Robert Bonomo)	Not sequenced	0.25	I	1	I	>64	R	64	R
VA-Ab91	Case Western Reserve University (Dr. Robert Bonomo)	Not sequenced	0.13	I	1	I	>64	R	64	R
VA-Ab92	Case Western Reserve University (Dr. Robert Bonomo)	Not sequenced	0.13	I	1	I	>64	R	64	R
VA-Ab97	Case Western Reserve University (Dr. Robert Bonomo)	Not sequenced	1	I	0.5	I	64	R	0.5	S
